# Shorter Maintenance Therapy in Childhood Acute Lymphoblastic Leukemia: The Experience of the Prospective, Randomized Brazilian GBTLI ALL-93 Protocol

**DOI:** 10.3389/fped.2016.00110

**Published:** 2016-10-17

**Authors:** Silvia R. Brandalise, Marcos B. Viana, Vitória R. P. Pinheiro, Núbia Mendonça, Luiz F. Lopes, Waldir V. Pereira, Maria L. M. Lee, Elitânia M. Pontes, Gláucia P. Zouain-Figueiredo, Alita C. A. C. Azevedo, Nilma Pimentel, Maria Z. Fernandes, Hilda M. Oliveira, Sônia R. Vianna, Carlos A. Scrideli, Fernando A. Werneck, Maria N. Álvares, Érica Boldrini, Sandra R. Loggetto, Paula Bruniera, Maria J. Mastellaro, Eni M. Souza, Rogério A. Araújo, Flávia Bandeira, Doralice M. Tan, Nelson A. Carvalho, Maria A. S. Salgado

**Affiliations:** ^1^Boldrini Children’s Center, Campinas, Brazil; ^2^Federal University of Minas Gerais, Belo Horizonte, Brazil; ^3^Onco Bahia Hospital, Salvador, Brazil; ^4^A. C. Camargo Hospital, São Paulo, Brazil; ^5^Santa Maria University, Santa Maria, Brazil; ^6^GRAACC, São Paulo, Brazil; ^7^GRUPO Hospital, São José, Brazil; ^8^Nossa Senhora da Glória Hospital, Rio de Janeiro, Brazil; ^9^Varela Santiago Children’s Hospital, Natal, Brazil; ^10^Erick Loeff Hospital, Salvador, Brazil; ^11^Felicio Rocho Hospital, Belo Horizonte, Brazil; ^12^State Public Hospital, São Paulo, Brazil; ^13^Clinic Hospital, Ribeirão Preto, Brazil; ^14^State Public Hospital, Rio de Janeiro, Brazil; ^15^Center Chemotherapy LTD., Belo Horizonte, Brazil; ^16^São Judas Tadeu Hospital, Barretos, Brazil; ^17^Hematology Center, São Paulo, Brazil; ^18^Santa Casa, São Paulo, Brazil; ^19^CLEMED Clinic, Jundiai, Brazil; ^20^Ana Costa Hospital, Santos, Brazil; ^21^Cancer Hospital, Uberlândia, Brazil; ^22^Hematology and Hemotherapy Foundation (HEMOPE), Recife, Brazil; ^23^St. Joachim’s Portuguese Charity Hospital, São Paulo, Brazil

**Keywords:** childhood acute lymphoblastic leukemia, maintenance ALL chemotherapy, pediatric ALL survival in middle-low-income countries

## Abstract

**Aim:**

Maintenance therapy is an important phase of the childhood ALL treatment, requiring 2-year long therapy adherence of the patients and families. Weekly methotrexate with daily 6-mercaptopurine (6MP) constitutes the backbone of maintenance therapy. Reduction in the maintenance therapy could overweight problems related with poverty of children with ALL living in limited-income countries (LIC).

**Objective:**

To compare, prospectively, the EFS rates of children with ALL treated according to two maintenance regimens: 18 vs. 24 months duration.

**Materials and methods:**

From October 1993 to September 1999, 867 consecutive untreated ALL patients <18 years of age were treated according to the Brazilian Cooperative Group for Childhood ALL Treatment (GBTLI) ALL-93 protocol. Risk classification was based exclusively on patient’s age and leukocyte count (NCI risk group) and clinical extra medullary involvement of the disease. Data were analyzed by the intention-to-treat approach.

**Results:**

Fourteen patients (1.6%) were excluded: wrong diagnosis (*n* = 7) and previous corticosteroid (*n* = 7). Of the 853 eligible patients, 421 were randomly allocated, at study enrollment, to receive 18-month (group 1) and 432 to receive 24-month (group 2) maintenance therapy. Complete remission rate was achieved in 96% of the patients (817/853). Twenty-eight patients (3.4%) died during the induction phase. Thirty-four patients (4.0%) were lost to follow-up. The overall EFS was 66.1 ± 1.7% at 15 years. No difference was seen according to maintenance: EFS_15y_ was 65.8 ± 2.3% (group 1) and 66.3 ± 2.3% (group 2; *p* = 0.79). No difference between regimens was detected after stratifying the analyses according to factors associated with adverse prognosis in this study (age group <1 year or >10 years and high WBC at diagnosis). Overall death in remission rate was 6.85% (56 patients). Deaths during maintenance were 13 in group 1 and 12 in group 2, all due to infection. Over 15 years of follow-up, two patients both from group 2 presented a second malignancy (Hodgkin’s disease and thyroid carcinoma) after 8.3 and 11 years off therapy, respectively.

**Conclusion:**

Six-month reduction of maintenance therapy in ALL children treated according to the GBTLI ALL-93 protocol provided the same overall outcome as 2-year duration regimen.

## Introduction

The last four decades have witnessed tremendous improvement on the survival rates of children suffering from acute lymphoblastic leukemia (ALL), which was made possible, thanks to the prominent effort of various multicenter Cooperative ALL Treatment Groups. Different immunophenotype and genetic subgroups of ALL were identified, which in conjunction with the evaluation of the patient’s initial response to therapy, through minimal residual disease (MRD) quantification at different time-points, have allowed the development of tailored treatment protocols by all the Cooperative Groups. Today, in the best contemporary treatment protocols, survival rates for childhood ALL patients are above 85%. Approximately one-third of all deaths are due to treatment toxicity, even with the high-quality supportive care available in high-income countries (1, 2).

However, for children with ALL living in low- and middle-income countries, survival rates are yet significantly lower than those attained in high-income areas of the world (3, 4). Several factors contribute to the inferior survival results, including lower socioeconomic status and education, limited access to specialized centers, reduced offer of genetic, immunophenotyping and molecular biology exams, and shortage of some chemotherapeutic drugs. All these factors are interconnected in a complex way and contribute to failure of adherence to treatment, thus playing an important role in clinical practice.

Maintenance therapy is as important as the more intensive and toxic earlier treatment phases, and often more challenging (5). Treatment of childhood ALL requires a prolonged maintenance phase that relies on self- or parent-administered daily antimetabolite chemotherapy given over a period of about 2 years (6–9). A systematic review of 42 randomized studies with 12,000 childhood ALL cases indicated that longer maintenance therapy gave a slightly lower risk of relapse but with no difference in survival due to a higher risk of death in remission (10). Lack of adherence, as well as associated infections, are important issues associated to unsuccessful maintenance treatment. We reasoned that a shorter maintenance therapy could be advantageous for the patients and their families, both by favoring adherence and by shortening the immunosuppression period. In the Brazilian Cooperative Group for Childhood ALL Treatment ALL-93 protocol (GBTLI ALL-93), the treatment schema included the induction (phase 1 and 2), consolidation and an intensification phase, followed by 18- or 24-month maintenance therapy.

### Objective

The primary aim of this study was to compare, according to the intention-to-treat, the event-free survival (EFS) rates in children with ALL randomized at diagnosis, to receive a maintenance therapy regimen of 18 vs. 24 months duration.

## Materials and Methods

### Patients

Eight hundred and sixty seven consecutive patients with newly diagnosed ALL, aged 0–18 years, who received no previous glucocorticoid treatment, were enrolled in the clinical trial GBTLI ALL-93 between October 1993 and September 1999. Twenty-five Brazilian institutions participated in the study. The diagnosis of ALL was based only on the morphological and cytochemistry features of the leukemic cells from bone marrow specimens. Immunophenotype and genetic features of the leukemic blasts were not required as obliged inclusion criteria, due to the lack of these exams in most Brazilian institutions at that time. Central morphological review was recommended. The protocol was approved by the Ethical Committee (IRB) of each of the 25 participating institutions and written informed consent was obtained for each participant (patients or parents/guardians, as appropriate).

Initial risk classification was based on patient’s age and leukocyte count at baseline (National Cancer Institute Risk Group Criteria) and clinical extra medullary involvement of the disease. As previously mentioned, immunophenotyping, ploidy, and cytogenetic were not considered as a risk variable in the study. Patient’s biological response was also not considered to define further risk stratification. Very low-risk group (VLR) was defined by age ≥1 and <10 years, with WBC ≤ 10,000/mm (3), no mediastinal mass or central nervous system (CNS) involvement, and hepatosplenomegaly <5 cm below costal margin. Low-Risk Group (LR) was defined by age ≥1 and <10 years, WBC > 10,000/mm^3^ and <50,000/mm^3^, and/or mediastinal mass, and/or hepatosplenomegaly ≥5 cm. High-Risk Group (HR) was defined by age <1 or ≥10 years, and/or WBC ≥ 50,000/mm^3^, and/or CNS involvement at diagnosis. Complete clinical examination was routinely performed at the time of enrollment into the study.

Only 14 patients were excluded at diagnosis for the following reasons: acute myeloid leukemia (AML) diagnosis in 7 children and previous corticosteroid administration in other 7 patients. Of the 853 eligible patients, 421 were randomly allocated to receive 18 months (group 1) and 432 to receive 24-month (group 2) maintenance therapy. Randomization was performed at study enrollement by a central office. Fifteen children did not follow the randomized group due to medical misunderstanding: 12 from the 18-month group and 3 from the 24-month group. It is important to emphasize that the overall treatment duration refers to the actual therapy received by the patient. Thirty-four patients were lost to follow-up (4.0%), being analyzed only till the abandonment time. For statistical analysis, abandonment was considered as an event.

### Treatment Schedule

Regimens were proposed according to the initially defined risk groups, as detailed in Tables 1 and 2. Briefly, VLR and LR group patients received *Induction therapy* (6 weeks) with dexamethasone (DEXA) 6 mg/m^2^/day × 28 days, Vincristine (VCR) 1.5 mg/m^2^/week × 4, Daunomycin 25 mg/m^2^/week × 4, l-Asparaginase 10,000 U/m^2^ IM × 8 and Ara-C 75 mg/m^2^/dose × 8 and triple intrathecal therapy (TIT) with methotrexate (MTX)/Cytarabine (Ara-C)/DEXA (according to age) at days 0, 29, and 43. *Intensification phase* (6 weeks) with MTX 2 g/m^2^ IV 24 h infusion with leucovorin (LCV) rescue 15 mg/m^2^/dose × 4, 6-Mercaptopurine (6-MP) 50 mg/m^2^/day × 6 weeks, and TIT × 4. *Reinduction phase* (6 weeks) with DEXA 6 mg/m^2^/day × 3 weeks, VCR 1.5 mg/m^2^/week × 4, l-ASP 10,000 U/m^2^ IM × 4, 6-MP 50 mg/m^2^/day × 2 weeks, Ara-C 75 mg/m^2^/day SC × 8 days, and TIT × 3. Patients were centrally randomized to receive *maintenance therapy* during 18 months (group 1) or 24 months (group 2) with 6-MP 50mg/m^2^/day continuously, MTX 25 mg/m^2^/week IM, and TIT each 8 weeks during all maintenance. *Pulses* with one single dose of VCR 1.5 mg/m^2^ and DEXA 4 mg/m^2^/day × 7 days were done each 8 weeks only during the first year of the maintenance treatment, for the LR Group of patients. Prophylactic CNS radiation was not performed in any LR.

**Table 1:** **Therapy for very low-risk and low-risk ALL patients on GBTLI ALL-93 protocol**.

Phase	Length
Induction, first phase	4 weeks
Dexametasone 6 mg/m^2^/d orally × 28 days	
Vincristin 1.5 mg/m^2^/dose IV (maximum 2 mg); days 1, 8, 15, and 22	
Daunomycin 25 mg/m^2^/dose IV (1 h inf.); days 1, 8, 15, and 22	
TIT^a^ at days 1 and 29	
Induction, second phase	2 weeks
Each 2 days, start day 29	
l-Asparaginase 10,000 IU/m^2^/dose IM (1 h inf.) × 8 doses	
Cytarabine 75 mg/m^2^/d SC × 4 doses weekly; days 29–32 and 40–43	
TIT^a^ at day 43	
Intensification	6 weeks
Each 2 weeks	
Methotrexate 2 g/m^2^ IV (24 h inf.) with	
LCV rescue 15 mg/m^2^/dose at hours 36, 42, 48, and 54	
6-Mercaptopurine 50 mg/m^2^/d orally × 6 weeks	
TIT^a^ each 2 weeks after systemic MTX infusion (×4)	
Reinduction, first phase	4 weeks
Dexametasone 6 mg/m^2^/d orally × 21 days	
Vincristin 1.5 mg/m^2^/dose IV (maximum 2 mg); days 106, 113, 120, and 127	
l-Asparaginase 10,000 IU/m^2^/dose IM × 4 doses; days 106,109,113, and 116	
TIT^a^ at day 106 and 126	
Reinduction, second phase	2 weeks
6-Mercaptopurine 50 mg/m^2^/d orally × 14 days	
Cytarabine 75 mg/m^2^/d SC × 4 doses weekly; days 134–137 and 145–148	
TIT^a^ at days 134, 141, and 148	
Maintenance therapy randomization	18 or 24 months								
GROUP 1 and GROUP 2									
6-MP 50 mg/m^2^/d orally									
MTX 25 mg/m^2^/dose IM weekly									
TIT each 8 weeks									
For low-risk group, pulses every 8 wk									
DEXA 4 mg/m^2^ every other day × 3									
VCR 1.5 mg/m^2^ IV (maximum 2 mg) at day 1									

*^a^Dose according to age*.

*GBTLI, Brazilian Childhood Cooperative Group for ALL Treatment; ALL, acute lymphoblastic leukemia; TIT, triple intrathecal chemotherapy (methotrexate, cytarabine, and dexamethasone); IV, intravenous; IM, intramuscular; SC, subcutaneous; Inf., infusion; 6MP, 6-mercaptopurine; MTX, methotrexate; VCR, vincristine; LCV, leucovorin; d, day; h, hour; w/, with; wk, week*.

**Table 2:** **Therapy for high risk ALL patients on GBTLI ALL-93 protocol**.

Phase	Length
Induction, first phase	4 weeks
Dexametasone 6 mg/m^2^/d orally × 28 days	
Vincristin 1.5 mg/m^2^/dose IV (maximum 2 mg); days 1, 8, 15, and 22	
Daunomycin 25 mg/m^2^/dose IV (1 h inf.); days 1, 8, 15, and 22	
l-Asparaginase 10,000 IU/m^2^/dose IM (1 h inf.) × 8 doses; days 15–22	
TIT^a^ at days 1, 15, and 29	
Induction, second phase	2 weeks
Cytarabine 750 mg/m^2^/d IV (3 h inf.) each 12 h × 6 doses; days 36–38	
l-Asparaginase rescue 6,000 IU/m^2^/dose IM × 6 doses at hour 6	
TIT^a^ at day 43	
Intensification	6 weeks
Each 2 weeks	
Methotrexate 2 g/m^2^ IV (24 h inf.) with	
LCV rescue 15 mg/m^2^/dose at hours 36, 42, 48, and 54	
6-Mercaptopurine 50 mg/m^2^/d orally × 6 weeks	
TIT^a^ each 2 week after systemic MTX infusion (×4)	
Reinduction, first phase	4 weeks
Dexametasone 6 mg/m^2^/d orally × 21 days	
Vincristin 1.5 mg/m^2^/dose IV weekly (maximum 2 mg); days 106, 113, 120, and 127	
l-Asparaginase 10,000 IU/m^2^/dose IM × 4 doses; days 106,109,113, and 116	
TIT^a^ at day 106 and 126	
Reinduction, second phase	2 weeks
6-Mercaptopurine 50 mg/m^2^/d orally × 14 days	
Cytarabine 75 mg/m^2^/d SC × 4 doses weekly; days 134–137 and 145–148	
TIT^a^ at days 134, 141, and 148	
CNS RT^a^	
Maintenance therapy, first phase (weeks 23–77)	18 or 24 months																
Block A (6 blocks) weeks 23, 32, 41, 50, 59, and 68																	
Cytarabine 750 mg/m^2^/d IV (3 h inf.) each 12 h × 6 doses; days 36–38																	
l-Asparaginase rescue 6,000 IU/m^2^/dose IM × 6 doses at hour 6																	
Block B																	
DEXA 3 mg/m^2^/d orally × 21 days																	
VCR 1 mg/m^2^ IV (maximum 2 mg) at days 1,8, and 15																	
Block C																	
6-MP 75 mg/m^2^/d orally × 21 days																	
MTX 40 mg/m^2^/dose IM; days 1, 8, and 15																	
Maintenance therapy, second phase																	
Pulses every 8 wk (start week 77 until the week 103, for group 1 or the week 130, for group 2)																	
DEXA 4 mg/m^2^/d orally × 7 days																	
VCR 1.5 mg/m^2^ IV (maximum 2 mg) at day 1																	
6-MP 50 mg/m^2^/d orally																	
MTX 25 mg/m^2^/dose IM weekly																	
TIT^a^ each 8 weeks, except for CNS radiated patients																	

*^a^Dose according to age*.

*GBTLI, Brazilian Childhood Cooperative Group for ALL Treatment; ALL, acute lymphoblastic leukemia; TIT, triple intrathecal chemotherapy (methotrexate, cytarabine, dexamethasone); IV, intravenous; IM, intramuscular; SC, subcutaneous; Inf., infusion; 6MP, 6-mercaptopurine; MTX, methotrexate; VCR, vincristine; LCV, leucovorin; d, day; h, hour; w/, with; wk, week; CNS, central nervous system; RT, radiation therapy*.

Patients of the HR Group received the *induction therapy*, as for the low-risk patients, with additional high-dose Ara-C 750 mg/m^2^ IV 3 h infusion each 12 h × 6 doses beginning at day 36, and l-ASP rescue at a dose of 6,000 U/m^2^ IM. *Intensification phase* was the same as for the low-risk group. *Reinduction phase* as for the low-risk group, except for the prophylactic CNS irradiation with 18 Gy (CNS-1 and CNS-2 patients) or 24Gy (CNS-3 patients). *Maintenance therapy* with rotating blocks A, B, and C from week 23 till week 77 (Block A = Ara-C 750 mg/m^2^ IV 3 h infusion each 12 h × 6 doses and l-ASP 6,000 U IM/m^2^ 6 h after the last Ara-C dose; Block B = DEXA 3 mg/m^2^/day × 21 days and VCR 1.0 mg/m^2^/week × 3, and Block C = 6-MP 75 mg/m^2^/day × 3 weeks and MTX 40 mg/m^2^/week IM × 3). After week 77, patients received daily 6-MP 50 mg/m^2^ continuously and MTX 25 mg/m^2^/week IM and pulses of one single dose of VCR 1.5 mg/m^2^ and DEXA 4 mg/m^2^/day × 7 were prescribed each 8 weeks, either until week 103 (group 1) or week 130 (group 2). Full dose chemotherapy was recommended with WBC ≥ 2,000/mm^3^, total phagocytes ≥500/mm^3^, and platelets counts ≥100,000/mm^3^.

It is important to emphasize that the overall treatment duration refers to the actual therapy received by the patient. Periods without programed chemotherapy were compensated.

## Statistical Analysis

Kaplan–Meier curves were used to illustrate children’s overall or EFS, and log rank tests to compare the curves for distinct groups of children. Overall survival (OS) was defined as the time period from diagnosis to death. EFS was defined as the time from diagnosis of ALL until the date of either induction failure, relapse, death in remission from any cause, the development of a second cancer, or until the date of last contact for all event-free survivors. Thirty-four patients (4.0%) were lost to follow-up: 12 children who were lost of follow-up after cessation of therapy (5.3 years of mean EFS), 9 during the maintenance phase (mean EFS 0.3 years), and 2 without reaching remission (EFS = 0). Maintenance EFS (M-EFS) was defined as the time from the beginning of the maintenance phase to relapse or death. Comparison between groups was done based on the intention-to-treat. The significance level was set at *p* ≤ 0.05. Statistical analyses were performed with the SPSS 20.0 software (SPSS Inc., Chicago, IL, USA) on the basis of data obtained up to June, 2016.

## Results

From October 1993 to September 1999, 867 consecutive patients from 25 Brazilian Institutions entered the study. Only 14 patients (1.6%) were excluded from the analysis for the following reasons: AML diagnosis (7 patients) and previous corticosteroid use (7 patients). In total, 853 patients were analyzed, being 406 of them classified as HR (48%). Clinical and laboratorial data are listed in Table 3. After induction therapy, 817 children (96%) achieved clinical complete remission (CCR). Thirty-six patients (4%) had induction failure. Twenty-eight children died during this initial phase (3.4%), mainly due to infections. Thirty-four patients (4.0%) were lost to follow-up. The mean time of follow-up for children without an event was 12.8 years (interquartile range: 5.7 years). Any event was registered in 285 patients. For these children, the median time to an event was 1.9 years (interquartile range 2.6 years). Clinical outcomes are summarized in Tables 4 and 5. Over 15 years of follow-up, 2 patients both from group 2 presented a second malignancy (Hodgkin’s disease and thyroid carcinoma) after 8.3 and 11 years of therapy, respectively.

**Table 3:** **Clinical and laboratorial data of 853 patients with ALL treated with GBTLI ALL-93 protocol**.

	No. of cases	%
Registered patients	867	100
Excluded patients	14	1.6
AML diagnosis	7	
Previous corticosteroids use	7	
Total of analyzed patients	853	98.4
White	627	73.5
Non-white	226	26.5
Age (in years)		
<1	23	2.7
≥1 to <10	636	74.6
≥10	194	22.7
Gender		
Male	437	51.2
Female	416	48.7
WBC (/mm^3^)		
<10,000	378	44.3
≥10,000 to <50,000	252	29.5
≥50,000 to <100,000	94	11.0
≥100,000	129	15.1
Risk group		
Very low risk	154	18.0
Low risk	293	34.3
High risk	406	47.6
CNS involvement at Dx	23	2.7
Testis involvement at Dx	8	0.9
Immunophenotype test		
T-ALL	76	12.8
B-ALL	517	87.2
Not performed	197	
Not referred	63	
Calla antigen (CD10) positive	475	85.3
Calla antigen (CD10) negative	82	14.7
Not performed	296	
Cytogenetic		
Exam performed	57	6.7
Normal	27	47.4
Hyperdiploidy	11	19.3
Hypodiploid	3	5.3
Not attained metaphases	16	28.0
Not performed	796	93.3
Molecular biology		
Exam performed	91	10.6
Chromos without abnormalities analyzed	66	72.5
Chromos abnormalities	25	37.8
*t* (12; 21)	15	
*t* (1; 19)	5	
*t* (4; 11)	1	
*t* (9; 22)	4	
Not performed	762	89.3

**Table 4:** **Clinical outcomes of 853 ALL patients treated according to GBTLI ALL-93 protocol**.

	No. of cases	%
Total number of analyzed patients	853	100
Attained remission at the end of induction	817	96
Induction failure (include induction deaths)	36	4
In CCR	590	69.1
Blast D8 (/mm^3^)		
<1,000	751	88.0
≥1,000	39	4.6
Not performed	63	
WBC D8 (/mm^3^)		
<10,000	766	89.8
≥10,000 and <50,000	23	2.7
≥50,000 and <100,000	3	0.3
≥100,000	0	
Not performed	61	
Site of relapses		
BM	138	16.1
CNS	12	1.4
Others	6	0.7
Combined	15	1.7
Death		
In induction	28	3.3
In remission	56	6.7
After relapse	154	18.0
Not remission	6	0.7
After BMT	1	0.1
Lost of follow-up	34	4.0
Mean of follow-up	9.1 years	

**Table 5:** **Treatment results according to clinical and laboratorial data of 853 ALL patients treated with the GBTLI ALL-93 protocol**.

		EFS at 15 years	95% confidence interval	*p* Value
Sex				
Male	437	0.662	0.617–0.707	0.98
Female	416	0.659	0.612–0.706	
Age				
<1 year	23	0.304	0.116–0.492	<0.00001
≥1 to <10 years	636	0.706	0.670–0.743	
≥10 years	194	0.554	0.483–0.624	
WBC (×10^9^/L)				
<10 × 10^9^/L	378	0.721	0.674–0.768	<0.00001
10−50 × 10^9^/L	252	0.676	0.617–0.734	
50−100 × 10^9^/L	94	0.563	0.463–0.664	
≥100 × 10^9^/L	129	0.527	0.440–0.612	
WBC (×10^9^/L)				
<50 × 10^9^/L	630	0.703	0.666–0.740	<0.00001
≥50 × 10^9^/L	223	0.542	0.477–0.607	
Immunophenotype^^a^^[Table-fn tfn3]				
Pre-B CD10 positive	473	0.731	0.690–0.772	<0.00001
Pre-B CD10 negative	44	0.475	0.327–0.623	
T-cell	76	0.460	0.348–0.572	
NCI risk groups				
Standard risk	447	0.773	0.732–0.814	<0.00001
High risk	406	0.538	0.489–0.587	
GBTLI risk group				
Very low risk	154	0.787	0.721–0.853	<0.00001
Low risk	293	0.765	0.715–0.816	
High risk	406	0.538	0.489–0.587	
CNS status^^a^^				
Positive	23	0.566	0.364–0.768	0.17
Negative	830	0.668	0.635–0.701	
Testicular involvement				
Yes	8	0.750	0.450–1.000	0.75
No	429	0.660	0.615–0.705	
Mediastinal involvement				
Yes	54	0.572	0.439–0.705	0.08
No	799	0.667	0.634–0.700	
D8 peripheral WBC^^a^^				
<5 × 10^9^/L	703	0.693	0.658–0.728	<0.00001
≥5 × 10^9^/L	89	0.493	0.389–0.597	
D8 peripheral blast^^a^^[Table-fn tfn3]				
Positive	182	0.531	0.457–0.605	<0.00001
Negative	608	0.713	0.676–0.750	

*^a^There are missing values for some children*.

**Figure 1:**
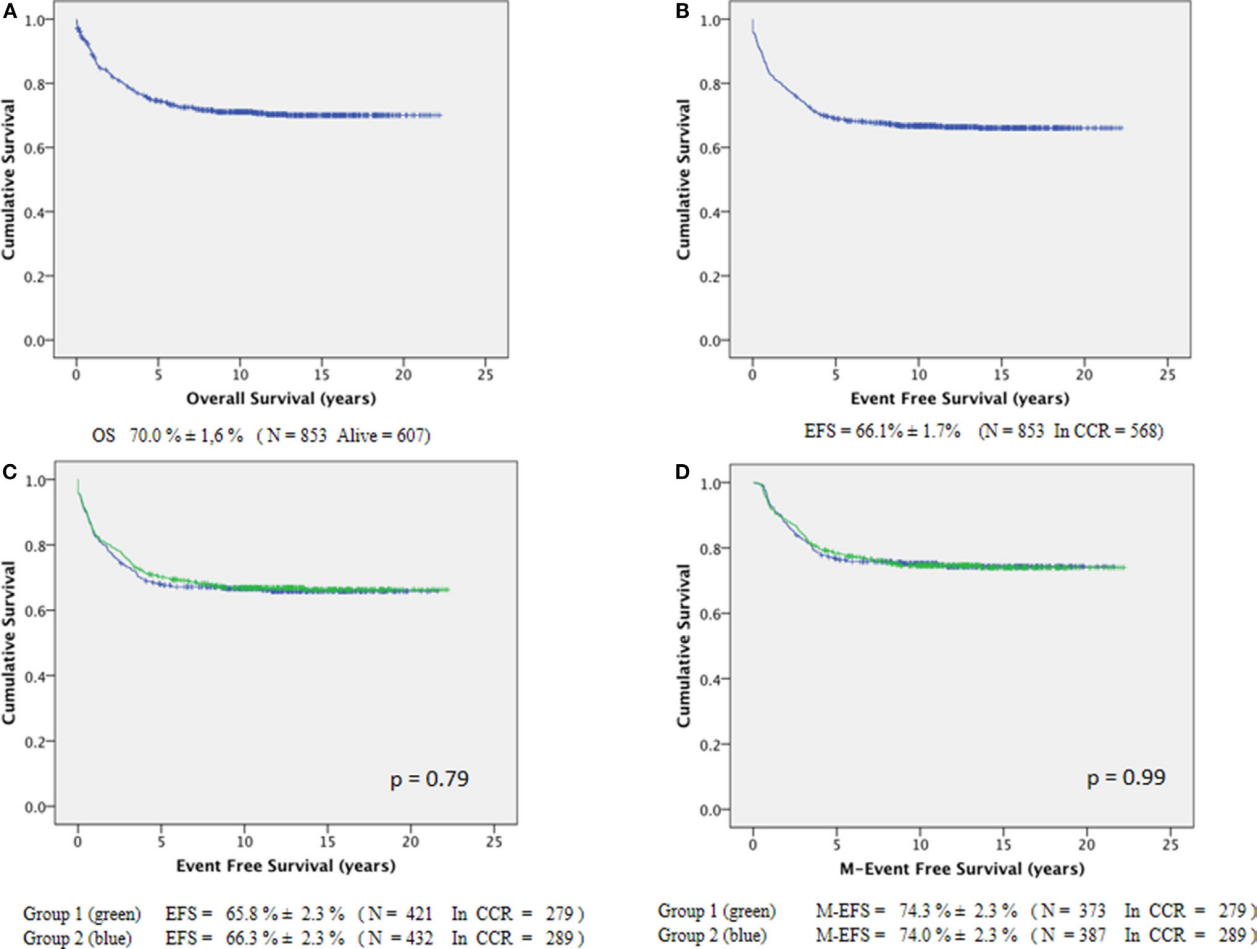
**Long-term survival of 853 ALL-children treated by GBTLI ALL-93 protocol: (A) overall survival, (B) event-free survival, (C) EFS according to maintenance regimen length, (D) M-EFS according to maintenance regimen length**.

The long-term 15-year OS was 70.0 ± 1.6% (Figure 1A). The overall EFS rate was 66.1 ± 1.7% (Figure 1B). No difference was detected according to the two maintenance regimens: group 1 (18-month duration) with pEFS_15y_ = 65.8 ± 2.3% and group 2 (24-month duration) with pEFS_15y_ = 66.3 ± 2.3% (*p* = 0.79; Figure 1C). Furthermore, even after stratifying the analyses according to the risk groups, there were no statistically significant differences in the EFS of group 1 vs. group 2, either among the very low and low-risk patients (*p* = 0.33), or among high-risk patients (*p* = 0.62). The same observation holds true relatively to gender (*p* = 0.60 and *p* = 0.87 for males and females, respectively), to age group (<1 year, ≥1 < 10 years, and ≥10 years), and WBC at diagnosis (< or ≥50,000/mm^3^). If only children who started the maintenance phase were considered (*n* = 760, 373 from 18-month group, and 387 from the 24-month group), excluding those who relapsed or died before it, the M-EFS was 74.3 ± 2.3% for group 1 and 74.0 ± 2.3% for group 2 (*p* = 0.99; Figure 1D).

The overall death in remission rate was 6.8% (56 patients). Importantly, of the 25 deaths that happened during the maintenance phase, 13 were from group 1 and 12 from group 2, all of them due to infections.

**Figure 2:**
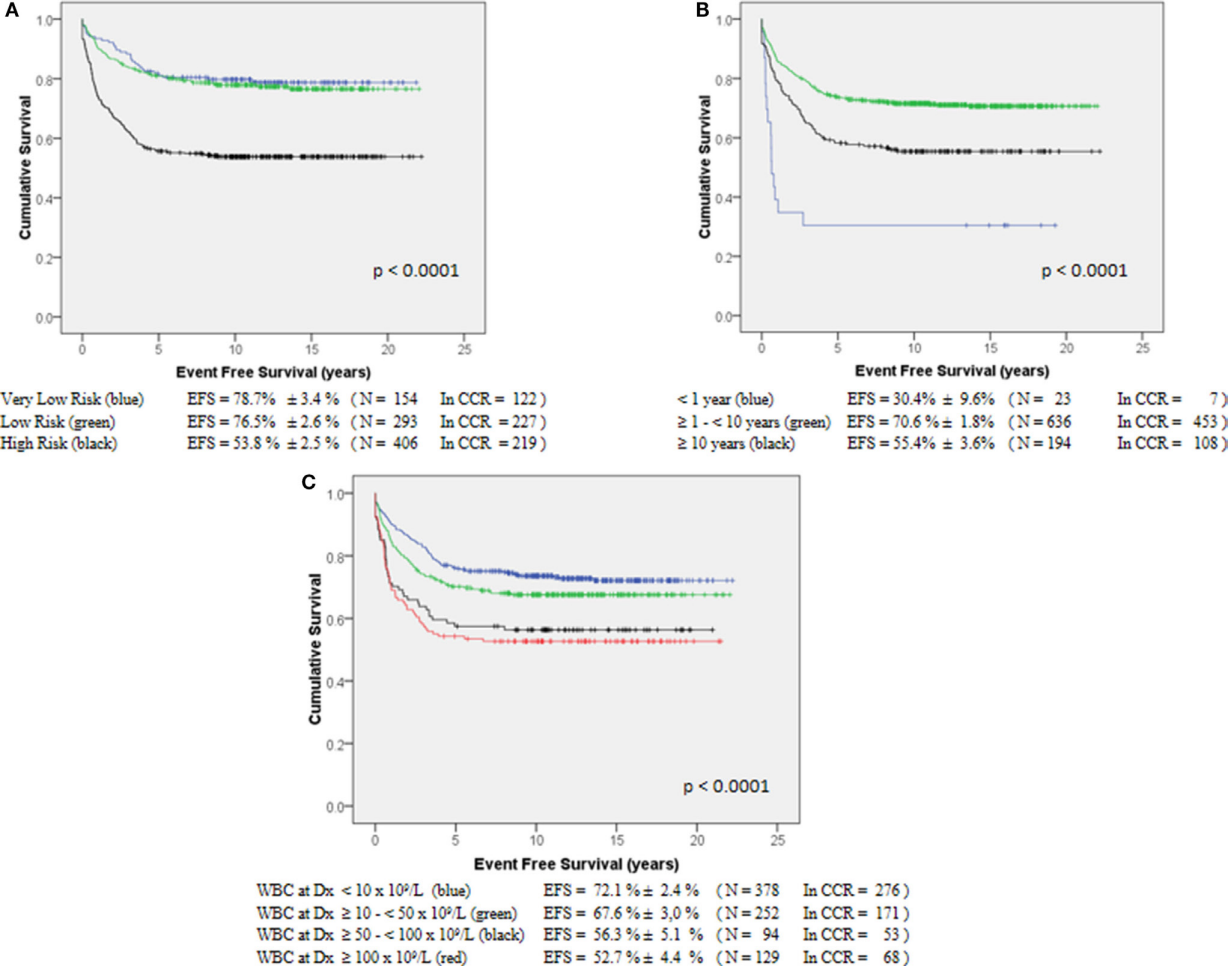
**Event-free survival of 853 ALL-children treated by GBTLI ALL-93 protocol: (A) according to the protocol risk factors, (B) according to age at diagnosis, (C) according to WBC count at diagnosis**.

According to the protocol risk group, the pEFS_15yr_ was 78.7 ± 3.4% (VLR group, *n* = 154), 76.5 ± 2.6% (LR, *n* = 293), and 53.8 ± 2.5% (HR Group, *n* = 406) (Figure 2A, *p* < 0.0001). According to age, children <1-year-old (*n* = 23) had a pEFS_15yr_ of 30.4 ± 9.6%; those >10-year-old (*n* = 194) had a pEFS_15y_ of 55.4 ± 3.6%, and those ≥1 and <10 years of age (*n* = 636) had a pEFS_15yr_ of 70.65 ± 1.8% (Figure 2B, *p* < 0.0001). Patients with initial WBC counts ≤10,000/mm^3^ (*n* = 378) had better pEFS_15yr_ rates as compared to those with ≥100,000/mm^3^ (*n* = 129): 72.1 ± 2.4 vs. 52.7 ± 4.4%, respectively (Figure 2C, *p* < 0.0001).

Only 593 patients (69.5%) had immunophenotypic analysis performed at diagnosis: 12.8% were T-cell ALL (*n* = 76), 7.4% were pre-B CD10 negative (*n* = 44), and 79.8% pre-B CD10 positive (*n* = 473). The pEFS_15yr_ for these three groups were 46.0 ± 5.7%, 47.5 ± 7.6%, and 73.1 ± 2.1%, respectively (*p* < 0.0001). Only 57 patients (6.7%) had cytogenetic and 91 patients (10.7%) had molecular biology studies performed.

**Figure 3:**
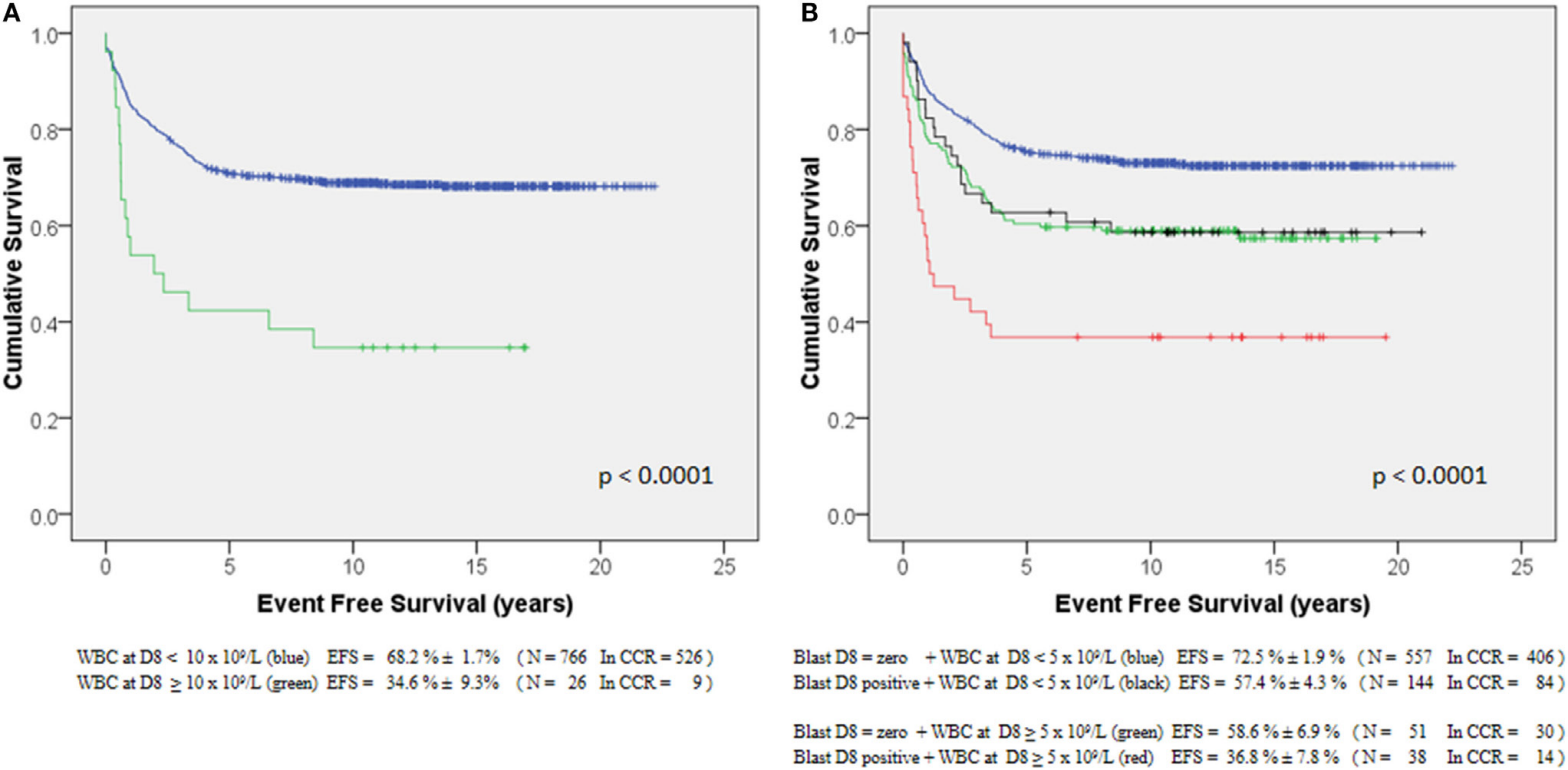
**Event-free survival of 853 ALL-children treated by GBTLI ALL-93 protocol related to initial induction therapy response (at D8): (A) according to peripheral WBC counts, (B) according to the presence of any blast and WBC counts**.

Even though initial response to therapy was not used for further children’s allocation into the risk groups, patients with peripheral WBC counts <10,000/mm^3^ at day 8 (D8) (*n* = 766) had long-term EFS of 68.2 ± 1.7%, while for those with ≥10,000/mm^3^ (*n* = 26) the EFS was 34.6 ± 9.3% (*p* < 0.0001) (Figure 3A). Similar results were observed in patients with D8 WBC < 5,000/mm^3^ (*n* = 703) when compared to those with ≥5,000/mm^3^ (*n* = 89): EFS = 69.3 ± 1.8 vs. 49.3 ± 5.3%, respectively (*p* < 0.0001). In addition, patients with any peripheral blasts at D8 (*n* = 182) had a worse prognosis than those with negative blasts (*n* = 608; *p* < 0.0001). Combined analysis revealed that patients with D8 WBC ≥ 5,000/mm^3^ and any blasts at D8 had a significantly poorer pEFS_15yr_ than patients with D8 WBC < 5,000/mm^3^ and no blast at D8 (*p* < 0.0001) (Figure 3B).

## Discussion

The GBTLI started the first trial in 1980. Consecutive studies ALL-82 and ALL-85 contributed to significant improvement of the survival cure rates for the Brazilian children and adolescents up to 70 ± 4% of EFS (11). Results here presented for the GBTLI ALL-93 (EFS_15y_ = 66.1 ± 1.7%) are comparable to both our earlier results (GBTLI ALL-85) and those obtained in other prospective treatment protocols for childhood ALL conducted by international cooperative groups in HICs over a similar period of time (1981–2000): UKALLX1 (EFS_5yr_ = 63.1 ± 2.2%) (12), COALL-92 (EFS_5yr_ = 76.9 ± 1.9%) (13), DCLSG-ALL-8 (EFS_5yr_ = 73 ± 2%) (14), EORTC-CLG 58881 (EFS_5yr_ = 70.9 ± 1.1%) (15), NOPHO ALL92 (EFS_5yr_ = 77.6 ± 1.4%) (16), BFM-95 ALL (EFS_5y_ = 79.6%) (17, 18), and AIEOP-95 (EFS_5y_ = 75.9%) (19).

It is difficult to compare the GBTLI ALL-93 protocol results with other contemporary published reports from the literature, considering that those studies included different ages, clinical, and laboratorial (immunophenotyping, cytogenetic, and ploidy) criteria for the group risk definitions. At that time, only age, WBC, and clinical extra medullary involvement of the disease were defined as risk criteria in our studies. Brief reports of our results have already been presented (20). Additionally, it is known that racial, nutritional, and socioeconomic variables also influence the survival of pediatric patients with acute leukemia (21–24).

Survival for children and adolescents with ALL has improved over time due to more precise risk classification and refinement of post-induction therapy through serial clinical trials (25). As one induction intensification and one or two consolidation therapies have improved cure rates of this disease, the necessity of several years of maintenance therapy has been recently questioned (5). As already mentioned, a systematic review of several randomized studies on childhood ALL, indicates that longer maintenance therapy did not improve survival because the somewhat lower risk of relapse was counterweighed by a higher risk of death in remission (10). Furthermore, longer as well as higher 6MP/MTX doses have, in three recent studies, been associated with an increased risk of second malignancies (6, 7, 26). In the GBTLI ALL-93 study, two second malignancies were registered in children from group 2 (24-month maintenance).

The long-term clinical results of the GBTLI ALL-93 protocol showed that it was feasible to shorten to only 18 months, the maintenance therapy for ALL patients, in the Brazilian setting. It remains to be seen if the modern protocols that reached survival rates above 85% depend upon the duration of the maintenance therapy. The lower survival rate in our study was mainly due to induction mortality and death in remission, predominantly, because of infections. Also, it is important to emphasize that in order to “overcome” the lack of diagnostic tools, the post-induction therapy was much more aggressive for the high-risk patients of the study, mainly because the high-dose Ara-C (750 mg/m^2^ × 6 doses) starting on day 36, shortly after obtaining the remission. Twenty-three patients died before attaining the maintenance period, 17 of them belonging to the HR.

Whether these patients who died due to toxicity and/or infections would benefit from a longer maintenance, has yet to be addressed. However, the fact that the next GBTLI ALL-99 protocol has already adopted the shorter maintenance therapy (18-month duration) with reasonable survival rates (27) allows us to suggest that such reduction may generally be advantageous for the patients. In our experience, there is no clear benefit to prolong this phase, neither considering the gender nor the NCI risk criteria at diagnosis. The possibility to decrease in 6 months, the treatment duration, contributes to more days at school, less expenditure with transportation to the clinics, and probably better quality of life. However, there is evidence that shortening the maintenance therapy too much may be risky. A previous study that shortened the total protocol duration from 2 to 1.5 years, significantly reduced the EFS. When all chemotherapy was limited to 52 weeks from diagnosis, the pEFS_5y_ was as low as 60%, even for non-high-risk ALL patients (28).

Even though early response evaluation to induction therapy was not used for patient’s stratification, D8 peripheral WBC counts were routinely recorded in the present study. Peripheral blast count reduction is well established by different investigators as a prognostic factor (17, 29). The GBTLI ALL-93 protocol proposed the simple WBC counts at D8 of treatment, as a predictor for therapy failure, based on previous data from our group showing that WBC counts were highly predictive of outcome (19, 30). The present study validated these earlier findings by showing that patients with either D8 peripheral WBC > 5,000 leukocytes/mm^3^ or the presence of any blast in D8 peripheral blood had adverse prognosis. Persistence of circulating blasts in peripheral blood at D8 after multi-agent chemotherapy in individual ALL patients had been previously reported as a poor prognostic factor with a relative risk of 2.9 (*p* < 0.0001) (31).

The rational of the study GBTLI ALL-93 was that shortening the maintenance therapy could increase survival both by reducing the immunosuppressive period and improving patient adherence. However, death in the maintenance phase was equally distributed between the two groups. Despite socioeconomic difficulties in Brazil at that time, only 4.0% of the patients were lost to follow-up. Lack of adherence to oral chemotherapy has been reported in children with ALL (32, 33), that was the reason to maintain, in this study, the MTX by IM route during all the maintenance therapy. Facilitators and barriers to adherence were not analyzed as part of the GBTLI ALL-93 study.

The GBTLI ALL-93 study offered a unique opportunity to address ALL treatment in the context of restricted access to modern technologies. Therapeutic strategies capable of overcoming these limitations in low- and middle-income countries remain an important topic to be investigated in the future, with controlled prospective studies performed in those parts of the world.

Unfortunately, recent national published data reveal a 5-year OS of only 47% for children with ALL living in different regions of Brazil. Those rates have not changed for a period of three decades (1996–2008) (34–36). Similarly, national published data from 1996 to 2008, covering different geographical regions of Brazil, reveal no changes in mortality rates due to leukemia in patients with <20 years of age. Surprisingly, differences between the rich and poor geographic areas of the country are lower than 2% (34). It is noteworthy that the survival rates of children with ALL living in the Rio Grande do Sul State reached EFS_5y_ = 62.41 ± 2.43% when treated with the GBTLI ALL protocols, while patients not included in any study had EFS_5y_ = 49.47 ± 4.15% (37). Unfortunately, less than 1% of the estimated new ALL patients with <18 years are registered in the GBTLI.

In 2015, there were 187 centers in Brazil registered at the Health Ministry for the care of children with cancer. Only 67 institutions were accredited for pediatric oncology care (38). How could children’s mortality rates due to cancer be reduced in low- and middle- income countries? Probably by: (1) limiting the number of specialized centers to deliver pediatric cancer treatment, (2) reinforcing and promoting institutional participation in cooperative prospective protocols with an overarching and monitoring structure, and (3) establishing a national health policy for accreditation and governance of childhood cancer treatment centers (3).

## Conclusion

Within the GBTLI ALL-93 protocol, the length of maintenance therapy could be safely abbreviated to 18 months, independently of the patient’s gender and risk group defined by age, WBC counts, and clinical extramedullary ALL involvement at diagnosis. It was feasible to achieve in Brazil long-term OS rates of 70% for children with ALL, corroborating the value of national prospective cooperative studies. The possibility of decreasing the maintenance treatment by 6 months may contribute to overcome major financial and cultural obstacles, remaining as adverse situations in middle- and low-income countries.

## Author Contributions

SB: conception and design, provision of study materials and patients, manuscript writing, revision, and final approval manuscript. MV: statistical analysis and interpretation, provision of study materials and patients, and manuscript revision. VP: provision of study patients. NM: provision of study patients. LL: provision of study patients. WP: provision of study patients. ML: provision of study patients. EP: provision of study patients. GZ-F: provision of study patients. AA: provision of study patients. NP: provision of study patients. MF: provision of study patients. HO: provision of study patients. SV: provision of study patients. CS: provision of study materials and patients and diagnosis analysis. FW: provision of study patients. MA: provision of study patients. EB: provision of study patients. SL: provision of study patients. PB: provision of study patients. MM: provision of study patients. ES: provision of study patients. RA: provision of study patients. FB: provision of study patients. DT: provision of study patients. NC: provision of study patients. MS: statistical analysis and interpretation, data manager.

## Conflict of Interest Statement

The authors declare that the research was conducted in the absence of any commercial or financial relationships that could be construed as a potential conflict of interest.
